# Nanoparticles in the environment: assessment using the causal diagram approach

**DOI:** 10.1186/1476-069X-11-S1-S13

**Published:** 2012-06-28

**Authors:** Suchi Smita, Shailendra K Gupta, Alena Bartonova, Maria Dusinska, Arno C Gutleb, Qamar Rahman

**Affiliations:** 1 Amity University, Lucknow Campus, Viraj Khand 5, Lucknow-226010, U.P., India; 2 CSIR-Indian Institute of Toxicology Research, Post Box 80, M.G. Marg, Lucknow-226001, U.P., India; 3 NILU - Norwegian Institute of Air Research POB 100, 2027 Kjeller, Norway; 4 Slovak Medical University, Department of Experimental and Applied Genetics, Limbova 12, 83303 Bratislava, Slovakia; 5 Department of Environment and Agro-biotechnologies (EVA), Centre de Recherche Public – Gabriel Lippmann, 4422 Belvaux, Luxembourg

## Abstract

Nanoparticles (NPs) cause concern for health and safety as their impact on the environment and humans is not known. Relatively few studies have investigated the toxicological and environmental effects of exposure to naturally occurring NPs (NNPs) and man-made or engineered NPs (ENPs) that are known to have a wide variety of effects once taken up into an organism.

A review of recent knowledge (between 2000-2010) on NP sources, and their behaviour, exposure and effects on the environment and humans was performed. An integrated approach was used to comprise available scientific information within an interdisciplinary logical framework, to identify knowledge gaps and to describe environment and health linkages for NNPs and ENPs.

The causal diagram has been developed as a method to handle the complexity of issues on NP safety, from their exposure to the effects on the environment and health. It gives an overview of available scientific information starting with common sources of NPs and their interactions with various environmental processes that may pose threats to both human health and the environment. Effects of NNPs on dust cloud formation and decrease in sunlight intensity were found to be important environmental changes with direct and indirect implication in various human health problems. NNPs and ENPs exposure and their accumulation in biological matrices such as microbiota, plants and humans may result in various adverse effects. The impact of some NPs on human health by ROS generation was found to be one of the major causes to develop various diseases.

A proposed cause-effects diagram for NPs is designed considering both NNPs and ENPs. It represents a valuable information package and user-friendly tool for various stakeholders including students, researchers and policy makers, to better understand and communicate on issues related to NPs.

## Background

Within HENVINET, an FP6 funded project, causal diagrams were developed as a tool to evaluate areas of agreement and disagreement between scientists and to identify gaps of knowledge [1,2]. The method of expert elicitation was applied by the HENVINET consortium to assess the health and policy implications of phthalates, where all details in the methodology behind the results presented here of the decaBDE and HBCD elicitations can be found [2]. In addition, an extensive review of the methodology with an overall discussion and analysis of the outcome for all the priority areas of the HENVINET consortium has been made [3]. Furthermore evaluations on advantages and disadvantages of the expert elicitation methodology have been made by others [4,5]. This approach has been chosen as one potential method to handle complex issues that are typically faced by the environment and health community and decision-makers. The current manuscript describes a proposed cause-effect diagram for nanoparticles (NPs) applicable to both naturally occurring NPs (NNPs) and man-made or engineered NPs (ENPs), and provides a short justification for the inclusion of the proposed elements into the presented cause-effect diagram. However, it has to be noted that the presented cause-effect diagram has not been the topic of an expert- elicitation yet.

At the moment, it is unclear whether the benefits of nanotechnologies outweigh the risks associated with environmental release and exposure to NPs and there are concerns that NPs can also lead to a new class of environmental hazards [6]. Until now, relatively few studies have investigated the toxicological and environmental effects of exposure to NPs and ENPs. However, there is enormous effort at national and at international levels including the OECD and the European Union to investigate the impact of NPs on the environment and health. No clear guidelines exist on how to evaluate and quantify these effects, the provision of systematic information following NPs from releases to effects was requested [7] and furthermore it was argued to apply an integrated approach [8]. NPs differ in size, shape, chemical composition and in many physico-chemical properties. It is therefore crucially important to know which properties may cause adverse health effects [9].

**Figure 1:**
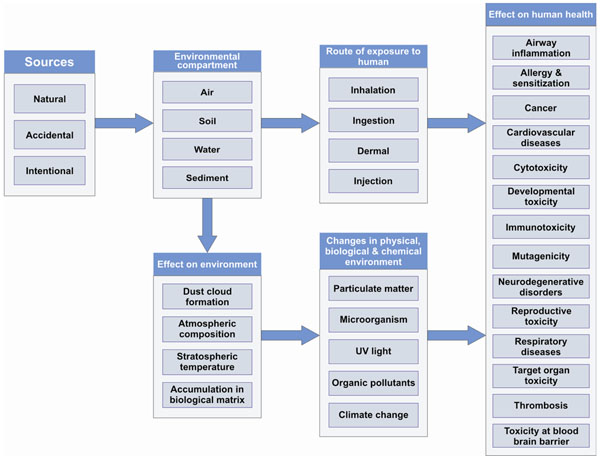
Proposed cause-effect diagram of NPs on environment and human health

Natural and engineered NPs present in the environment are influenced by a large number of physico-chemical processes and show different behaviour in organisms, soil, and water. The accumulation of engineered NPs (ENPs) has been shown in various organisms and environmental compartments, such as blue and green algae, fish and other aquatic organisms as well as soil and sediments [10-16]. Due to the low number of systematic studies and lack of knowledge on physicochemical properties and behaviour of NPs, these reports show an inconsistent picture of the effect of NPs on various environmental processes and their impact on human health. In the present work, we attempt to describe the elements of a cause-effect diagram as already developed within HENVINET for other environmental hazards and disease complexes [1,2]. The diagram for NPs is designed on the basis of current understanding of NPs mediated toxicity reports and review articles already available (Figure 1). These diagrams have been shown to be helpful to evaluate the level of confidence in the current ability of scientists to predict the magnitude of a disease burden that are expected to occur as a result of the release of NPs in the environment [1].

## Elements of the NP cause-effect diagram

### Sources of nanoparticles

Sources of NPs can be classified as natural or intentional and unintentional anthropogenic activities. NNPs exist in the environment since the beginning of Earth’s history and are common and widely distributed throughout the earths´ atmosphere, oceans, surface and ground water, soil and even in living organisms. Major natural processes that release NPs in the atmosphere are forest fires, volcanic activities, weathering, formation from clay minerals, soil erosion by wind and water, or dust storms from desert. Atmospheric dust alone is estimated to contain as much as several million of tons of natural NPs within a year [17]. Naturally occurring ambient NPs are quite heterogeneous in size and can be transported over thousands of kilometres and remain suspended in the air for several days.

**Figure 2:**
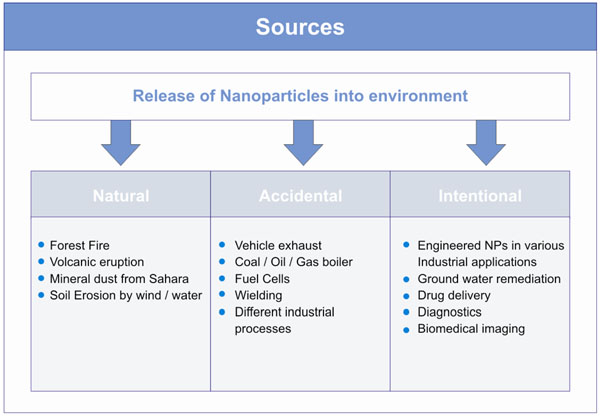
Common sources of NPs in the environment.

Man-made ENPs are unknowingly or purposely released in the environment during various industrial and mechanical processes (Figure 2). These NPs are very heterogeneous in nature and currently it is difficult to measure the impact on human health. The annual release of ENPs into the environment cannot be accurately estimated [6] while production volumes are strongly increasing [18]. The unfiltered exhaust gases from diesel engines contain large quantities of potentially harmful NPs from the incomplete combustion of fuel. In the fireplace at home, fullerenes like buckyballs or buckytubes are formed when wood is burned. In industrial processes, coal, oil, and gas boilers release tons of NPs unintentionally [19].

As a growing and widely applied science, nanotechnology has a global socioeconomic value, with applications ranging from electronics to biomedical uses [20]. With the advancement of industrial processes and nanotechnologies, a large number of ENPs are been manufactured and it is inevitable that during the use of the related products, ENPs are released in the air, water and soil both intentionally and unintentionally.

Because of their small size (less than 100 nm) and the very high surface to volume ratio, NPs usually display an enormously elevated reactivity potential. NPs can be assigned to a transitional range between single atoms or molecules and bulk material. The physicochemical features of NPs differ substantially from those of their respective bulk materials. Most of the ENPs are made up of carbon, silicon, metal or metal oxides and are believed to adversely affect the environment and human health directly or indirectly together with naturally occurring NPs [3]. Certain carbon nanotubes can cause the onset of mesothelioma, a type of cancer previously thought to be only associated with asbestos exposure, once inhaled [4,5,21]. However, this is not caused by the fact that nanotubes have two dimensions smaller than 100 nm but because they in fact interact with cells similarly to asbestos [4].

### Natural occurrence of NPs in environmental matrices and their effects

NNPs can serve as a model for ENPs in the environment and naturally occurring mineral NPs. Their behaviour can point out important mechanisms in which NPs can move through environments and affect various environmental systems [22]. Once NPs are released in the environment from either natural or man-made sources, very little is known about their environmental fate. Especially NNPs in the atmosphere have been studied in atmospheric sciences [23]. After release in the environment, NPs will accumulate in various environmental matrices such as air [23], water, soil and sediments including wastewater sludge [24-28].

### Effects of NPs on the environment

Various environmental processes that depend on the presence of physical entities are likely to be altered by the accumulation of NPs in the environment. Some of these processes are dust cloud formation, environmental hydroxyl radical concentration, ozone depletion, or stratospheric temperature change.

#### Effect of NNPs on dust cloud formation and decrease in sun light intensity

NNPs are thought to play an important role in dust-clouds formation after being released into the environment as they coagulate and form dust cloud [29]. 70% of the brown clouds over South Asia are made up of soot from the burning of biomass; largely wood and animal dung used for cooking and mainly contains particulate matters and carbon NPs from unprocessed fuel [30]. The regional haze, known as atmospheric brown clouds, contributes to glacial melting, reduces sunlight, and helps create extreme weather conditions that impact agricultural production. The pollution clouds also reduced the monsoon season in India [31,32]. The weather extremes may also contribute to the reduced production of key crops such as rice, wheat and soybean [29].

#### Asian brown clouds impact on Himalayan glaciers

Asian brown clouds carry large amounts of soot and black carbon which are deposited on the glaciers. This could lead to higher absorption of the sun's heat and potentially contributing to the increased melting of glaciers [30]. The Himalayan glaciers provide the source of many of Asia’s great rivers, with millions of people depending on them for food and water and because Asian brown clouds increase atmospheric temperature these glaciers have been decreasing over the past decades.

#### Asian brown clouds impact on agriculture

Dimming induced by atmospheric brown clouds is considered the major cause of the changing pattern of rainfall in Asia, with decreasing rainfall in some parts while other parts experience intense floods. Asian brown clouds are interfering with centuries old monsoon patterns with disastrous consequences for food production [29]. The large concentration of ozone in atmospheric brown clouds could decrease crop yields by as much as 20% [29,31].

#### Asian brown cloud impact on human health

**Figure 3:**
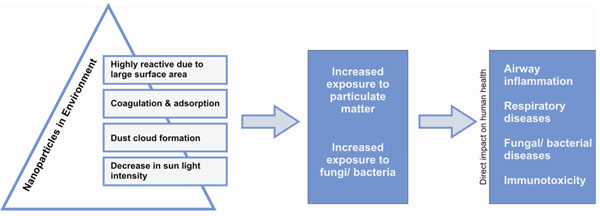
Impact of NPs on dust cloud formation and decrease in sunlight intensity and relation with various human health problems

A large part of the aerosol particles that make up atmospheric brown clouds are the result of the incomplete combustion of fossil fuels and bio-fuels. This increased exposure to particulate matter also increases the risk of exposure to pathogenic bacteria/ fungi [33,34]. The health impact of these particles is an increase in cardiovascular diseases, pulmonary illnesses, fungal/ bacterial diseases and chronic respiratory problems (Figure 3). The report estimates that in India and China alone, Asian brown clouds result in over 330,000 excess deaths per year mainly due to cardiopulmonary diseases [29].

#### Effect of NNPs on environmental hydroxyl radicals concentration and ozone depletion in the atmosphere

**Figure 4:**
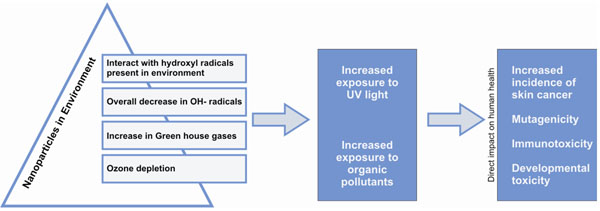
Schematic diagram how NPs are affecting the hydroxyl radical concentration, resulting in ozone depletion and human health problems

The hydroxyl radical, which is one of the most reactive free radicals in the environment and plays an important role in the photochemical degradation of natural organic matter and organic pollutants in the environment. NNPs being very reactive immediately bind with hydroxyl radicals and ultimately result in the overall reduction of hydroxyl radicals [35,36]. As hydroxyl radicals are strong oxidants and thereby degrading many pollutants, its reduction is responsible for the increase in green house gases, which are ultimately responsible for ozone layer depletion (Figure 4) and cause severe environmental damage [37]. Furthermore it increases the exposure to UV radiation [38], which leads to the increase in incidences of various types of skin cancer in humans.

#### Effect of NNPs on the decrease of environmental stratospheric temperature

**Figure 5:**
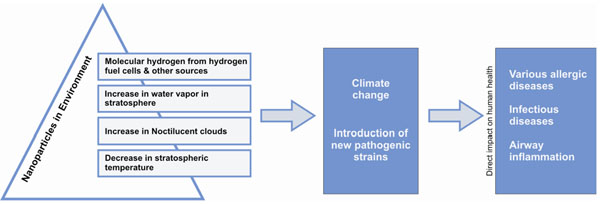
Effect of NPs on the increase in water vapour in stratosphere and decrease in stratospheric temperature resulting in various allergic diseases

NPs in the troposphere interact with molecular hydrogen accidentally released from hydrogen fuel cells and other sources [39,40]. Molecular hydrogen along with NPs moves up to the stratosphere, resulting in the abundance of water vapour in the stratosphere. This will cause stratospheric cooling, enhancement of the heterogeneous chemistry that destroys ozone, an increase in noctilucent clouds, and changes in tropospheric chemistry and atmosphere-biosphere interactions (Figure 5). Noctilucent clouds are composed of tiny crystals of water ice 40 to 100 nm in diameter and exist at a height of about 76 to 85 kilometres, higher than any other clouds in Earth's atmosphere. Similar to the more familiar lower altitude clouds, the noctilucent clouds are formed from water collecting on the surface of nano sized dust particles. The sources of both the dust and the water vapour in the upper atmosphere are not known with certainty. The dust is believed to come from micro meteors, although volcanoes and dust from the troposphere are also possibilities. The moisture could be lifted through gaps in the tropopause, as well as forming from the reaction of methane with hydroxyl radicals in the stratosphere. There is evidence that the relatively recent appearance of noctilucent clouds, and their gradual increase, may be linked to climate change [39].

### Accumulation of ENPs in selected biological matrices

**Figure 6:**
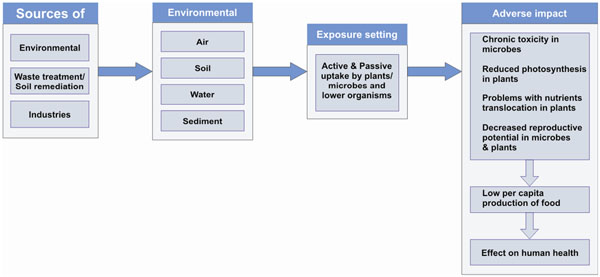
Exposure of NPs to plants, microbes and lower organisms resulting in adverse impact

It is inevitable that ENPs will be released into the soil and waters during their use and increase the load of ENPs in different environmental matrixes reflected by an increasing concern over the potential impact of ENPs in the environment on aquatic and terrestrial organisms [6,11,15,41]. Once released in the environment ENPs may enter plants and other microorganism by active or passive uptake [Figure 6]. NPs absorbed by microorganisms and plants, may enter into the food chain and cause serious alterations in humans and animals [42-44]. NPs due to highly reactive nature and large surface areas have potential to carry toxic materials, such as lipophilic pollutants and heavy metals [45].

**Figure 7:**
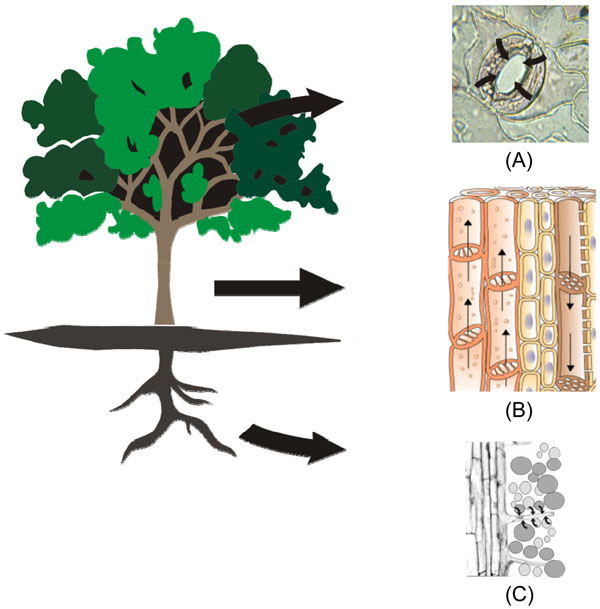
**Effect of NPs on plants** NPs may enter into the plants via (A) stomatal cells. NPs entered through stomata may deposit into the cellular system and can affect transpiration, plants respiration, photosynthesis. (B) NPs can deposit on sieve cells and interfere with the translocation of food material and block xylem cells. (C) NPs in soils may enter through the active or passive absorption by root hairs.

Some type of NPs may enter the plants via the root cell walls [Figure 7] [46-48]. Cell walls are semi-permeable and have pores with a size ranging from 5 to 20 nm that allow the passage of small particles. Thereby NPs and their aggregates with sizes smaller than the pore diameter may pass through the cell wall and reach the plasma membrane. There is some evidence that NPs may enter cells via embedded transport carrier proteins and ion channels and that they may interfere with normal metabolic processes, possibly by the production of reactive oxygen species (ROS) [11]. Airborne NPs accumulate over leaf surface and may enter into the cell through leaf stomata. Thus, plants with a high leaf area and stomatal indices may expect to have the higher interception potential for airborne NPs. Accumulation of NPs on stomatal tissues might alter the gas exchange; resulting in the foliar heating and adverse effects on plant physiology [49]. Carbon nanotubes and aluminium NPs have been identified to inhibit root growth in various economically important plant species by interacting with root surface [50,51]. Carbon black that aggregate on the sperm cells of a marine seaweed (*Fucus serratus*) were found to reduce the fertilization success rate [52]. Recent reports show the impact of NPs on various food crops. Carbon NPs diminished rice yields and made wheat more vulnerable to other pollutants [45,53], while again it has to be noted that this effect may be due to the asbestos-like behaviour of carbon nanotubes. Thus NPs are one of the major concerns for a future risk of low per capita food production. The accumulation of NPs on photosynthetic surfaces may cause shading effects, i.e. reduced sun light availability and hence reduced photosynthetic rate.

While the description of the ecotoxicity of NPs is not a central aim of this manuscript NP exposure related effects have been shown for a range of test organisms and NPs. TiO_2_ NPs were shown to adsorb on algal cell surface, resulting in the increase of cellular weight by more than 2 fold and affecting the algae’s ability to float and resulting in reduced sunlight availability for photosynthesis [11]. The toxicity of TiO_2_ NPs on green algae *Desmodesmus subspicatus* has been shown to be size dependent. Smaller NPs (~ 25 nm) showed a clear concentration-effect relationship (EC50 of about 40 mg/L), whereas the large particles (~ 100 nm) were found to be less toxic [10]. Silver NPs exerted considerable toxicity in a nematode (*Caenorhabditis elegans*), especially decreasing the reproduction potential and increased enzyme induction and protein formation [54] but have been shown to also affect a range of other organisms too [55]. NPs may impair the function or reproductive cycles of earthworms, which play a key role in nutrient cycling [56] hence possessing a hazard to induce ecological imbalances.

### Human exposure to nanoparticles

Exposure of humans to NPs mainly occur through natural routes (oral, pulmonary or skin uptake). Exposure assessment is difficult but necessary [8,57-59]. Furthermore many intentional processes such as medical applications may directly inject ENPs into the human body. Under practical conditions the most important routes of uptake for ENPs are inhalation or oral uptake [7], but this has not been specifically studied. More information is available for accidentally released NPs from combustion engines especially diesel exhaust [60,61]. In case of aerosolized silver-containing NPs that are widely used in consumer products due to their antimicrobial properties, environmental and human health risk were reviewed in detail [62]. NPs come in the direct contact with skin as they are widely used in various cosmetics and personal care products, and hence the assessment of toxicity due to dermal route of exposure is very critical [6][63][64]. While NPs are already present in food products such as ketchup, intake of NPs through food is another area where exposure assessment is crucial but very little information available on population exposures through ingestion [65]. To facilitate the toxicity assessment of NPs exposure to human, the establishment of exposure registries were recommended to enable the conduct of large-scale prospective multi-center epidemiologic studies [66].

### Human health impact of nanoparticles

**Figure 8:**
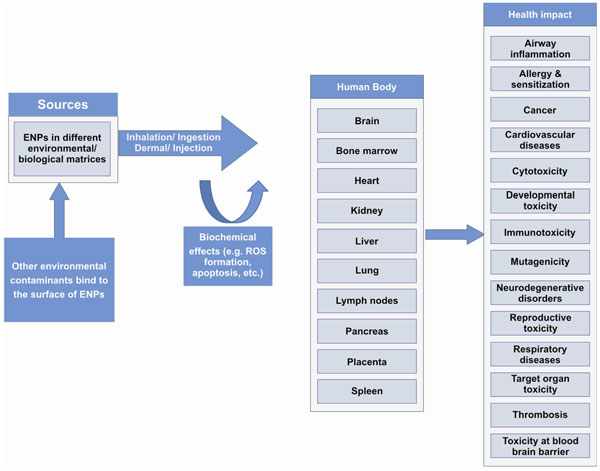
NPs exposure causes target organs toxicity by ROS formation and apoptosis resulting in adverse impact on human health

Change in the physical, biological and chemical component of the environment directly influences human health. Among them aggregation, agglomeration, dispersability, size, solubility, surface area, surface charge and surface chemistry/ composition have been identified to be most important parameters [9]. A number of potential health effects have been identified probably being related to the exposure of humans to ENPs (Figure 8).

Inhaled NPs are likely to evade phagocytosis, penetrate lung tissue, reaching interstitial spaces and enter blood circulation [67-69]. In the cardiovascular system platelet aggregation, and enhanced vascular thrombosis were observed [70]. Via the blood stream NPs can finally reach sensitive target sites such as lymph nodes, spleen, heart, kidney, liver, pancreas, bone marrow and brain [19,67,68,71-73]. Cell membrane penetration and particle accumulation in diverse cellular organelles (e.g. mitochondria) can finally lead to injurious responses within the crucial target organs and inflammation, immunotoxicity, cytotoxicity, genotoxicity and malignancy have been attributed to the nanoparticle-associated oxidative stress [18,21,74-77]. The oxidative stress resulting from the exposure to quartz and carbon black NPs can pose pronounced effects like interstitial fibrosis and airway inflammation [78-80].

## Conclusion

Nanotechnology, as a strongly growing and widely applied science, has a high potential of global socioeconomic value. On one hand, the new features of designed NPs provide unprecedented technical capabilities thereby enabling them to perform absolutely novel tasks in technology and science. Unfortunately, just the same new qualities can concurrently also include undesired intrinsic features, which sometimes lead to harmful interactions with exposed organisms.

In coherence with the described alarming aspects it seems to be a high time to establish linkages between direct and indirect health impact of NP exposure and evaluate the consensus among researchers and policy makers regarding the knowledge base. The causal diagram approach has proven to be a suitable conceptualization, simplification and visualization technique that allows communication linking the scientific disciplines involved, as documented by a wide range of examples [1,2,81,82]. In the near future it is envisaged to use this diagram as the basis for an internet-based tool for knowledge assessment. These causal diagrams provide an important platform to identify knowledge gaps and potential agreements or disagreements on the effect of NPs on various environmental processes and their impact on human health and can contribute to sustainable governance regarding the future use of NPs.

## Competing interests

None declared

## Authors' contributions

SS, SKG and QR conceived and designed the review, collected the data and drafted the manuscript. ACG, AB and MD commented and revised the draft manuscript and contributed with some sections. AB is HENVINET project coordinator and contributor to the framework development. All authors read and revised the final version of the manuscript.
